# Effects of Baduanjin Exercise on Rehabilitation of Patients With Mild to Moderate Parkinson’s Disease

**DOI:** 10.3389/fnins.2021.827180

**Published:** 2022-01-20

**Authors:** Shuangshuang Dong, Yiqing Wang, Hongyu Wei, Shouyun Du, Xiaojing Li, Jianbing Zhu, Yi Wang, Zenglin Cai

**Affiliations:** ^1^Department of Neurology, The Affiliated Suzhou Science and Technology Town Hospital of Nanjing Medical University, Suzhou, China; ^2^Department of Neurology, The First Affiliated Hospital of Kangda College of Nanjing Medical University, Lianyungang, China; ^3^Department of Neurology, Guanyun People’s Hospital, Lianyungang, China; ^4^Department of Radiology, The Affiliated Suzhou Science and Technology Town Hospital of Nanjing Medical University, Suzhou, China; ^5^Department of Neurology, Gusu University of Nanjing Medical University, Suzhou, China; ^6^Department of Neurology, Affiliated Lianyungang Hospital of Xuzhou Medical University, Lianyungang, China

**Keywords:** Parkinson’s disease, Baduanjin, motor symptoms, non-motor symptoms, gait

## Abstract

**Objectives:**

Rehabilitation, aerobic exercise, and many traditional Chinese exercises are known to significantly improve balance in patients with Parkinson’s disease. Baduanjin, a traditional physical and mental exercise, has long been practiced for health care as it regulates organs, the nervous and motor systems.

**Methods:**

We recruited 31 eligible participants. Patients underwent a 3-week Baduanjin program, including 35-min exercise daily. Scores on the Modified Unified Parkinson’s Disease Rating Scale (MDS-UPDRS), Non-motor Symptoms Scale (NMSS), and gait and balance tests were compared before and after the Baduanjin program.

**Results:**

MDS-UPDRS-total (*t* = 4.669, *P* ≤ 0.001), MDS-UPDRS part-I (*t* = 5.805, *P* ≤ 0.001), MDS-UPDRS part-II (*t* = 5.234, *P* ≤ 0.001), MDS-UPDRS part-III (*t* = 3.274, *P* = 0.003), and NMSS (*t* = 4.815, *P* ≤ 0.001) scores significantly decreased after the 3-week intervention. Gait parameters like step (*t* = 2.289, *P* = 0.030) and cycle (*t* = 2.181, *P* = 0.038) durations also significantly improved, while Balance-check^®^ indicators, including the total score (*t* = −2.147, *P* = 0.041) and grade (*t* = 3.432, *P* = 0.002) significantly differed before and after exercise.

**Conclusion:**

Baduanjin exercise shows beneficial effects for non-motor symptoms, balance, gait, and daily activities in patients with Parkinson’s disease. Baduanjin can be included in the patients’ family exercise, which is conducive to their rehabilitation, as well as for obtaining important social and economic benefits.

**Clinical Trial Registration:**

[www.ClinicalTrials.gov], identifier [ChiCTR-IPR-17011875].

## Introduction

Parkinson’s disease (PD) is the most common severe dyskinesia and the second most common neurodegenerative disease after Alzheimer’s, with a prevalence of 400–1,900 cases per 100,000 people worldwide (Pringsheim et al., 2015). The main pathological changes in PD involve the degeneration of the nigrostriatal dopaminergic pathway. Clinically, PD is characterized by resting tremor, bradykinesia, myotonia, and postural gait disorder (Poewe et al., 2017). In addition to motor symptoms, PD patients may also have non-motor symptoms, including sensory symptoms, autonomic nervous system defects, mental deficits, sleep disorders, etc. (Chaudhuri et al., 2017). Non-motor symptoms affect the quality of life of patients and sometimes even more than the motor symptoms (Chaudhuri et al., 2017).

For thousands of years, there have been many excellent traditional Chinese health exercises, such as Tai Chi, Qigong, and Baduanjin. Studies in the past decade have shown that Tai Chi and Qigong can improve a range of motor and non-motor symptoms associated with PD (Ni et al., 2014). Tai Chi and Qigong usually emphasize both standing and dynamic movements (such as pushing movements and raising gestures) that may affect gait, balance, and other functional activities. For these reasons, these two practices are currently considered a very effective intervention for PD (Peter and Crane-Godreau, 2013).

Baduanjin, one of the most common Chinese Qigong exercises, originates from the Song Dynasty and has existed for more than one thousand years (Liu et al., 2014). Because of its simplicity and easy learning, it is widely practiced by people of all ages. As a traditional physical and mental exercise, it has long been practiced for health reasons, as it regulates organs, the nervous system, and motor system (Chen et al., 2017). A number of studies have found that Baduanjin exerts positive effects on dyslipidemia (Mei et al., 2012), knee osteoarthritis (An et al., 2008), and hypertension (Chen et al., 2018). In addition, as a physical exercise, Baduanjin has also shown positive effects on psychological problems and suboptimal health status by improving cognitive function (Tao et al., 2017). Based on these findings, we speculated that Baduanjin might have positive effects in patients with PD. The purpose of the current study was to examine the effects of Baduanjin on motor and non-motor symptoms in patients who were diagnosed with mild to moderate PD.

## Materials and Methods

### Trial Design and Oversight

**FIGURE 1:**
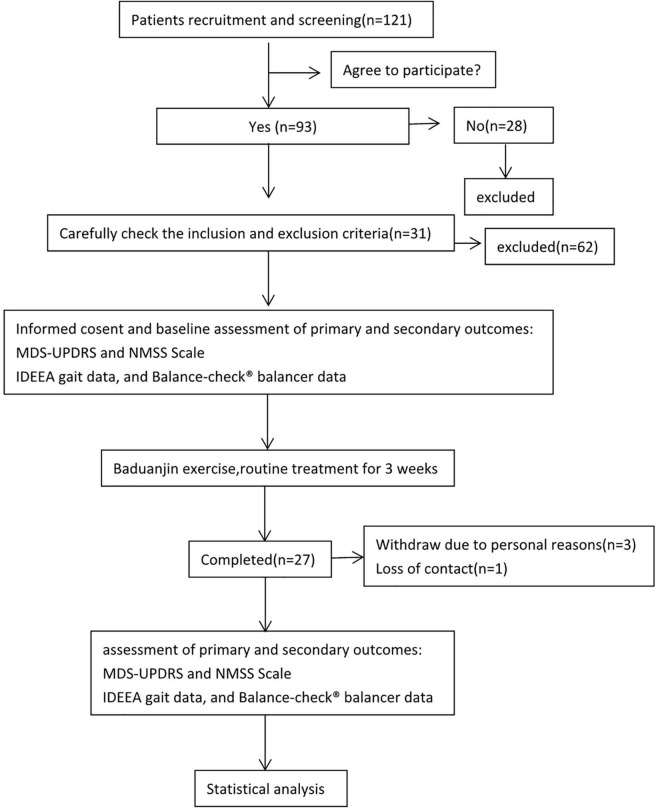
Flow diagram of the study. We recruited 121 patients with suspected Parkinson’s disease, 93 of whom agreed to be enrolled in the study, and 28 did not agree to enroll in the study. According to our enrollment criteria and exclusion criteria, 31 patients were finally included to participate in the study of Baduanjin exercise for 3 weeks. Among them, 3 patients withdrew midway, and 1 patient lost contact. Finally, the data of Modified Unified Parkinson’s Disease Rating Scale, Non-motor Symptoms Scale, gait, and balance tests of 27 patients before and after exercise were analyzed.

The study was a single-center, self-controlled trial conducted at Affiliated Lianyungang Hospital of Xuzhou Medical University, Lianyungang, China. The primary purpose was to evaluate the effect of Baduanjin on motor and non-motor symptoms in patients who were diagnosed with mild to moderate PD. A total of 31 eligible participants were recruited to our trial, starting from May 2017 to September 2018. Based on conventional medical treatment, participants were given a 3-week program of Baduanjin. Changes in scores on the Modified Unified Parkinson’s Disease Rating Scale (MDS-UPDRS) (Goetz et al., 2010) and Non-motor Symptoms Scale (NMSS) (Chaudhuri et al., 2010) from baseline to 3 weeks post-intervention served as primary outcome parameters, whereas changes in gait measured by The Intelligent Device for Energy Expenditure and Activity (IDEEA) and balance measured by Balance-check^®^ balancer served as the secondary outcome parameters. The study protocol and consent documents were reviewed and approved by the Ethics Committee of Nanjing Medical University [No: (2016)13], and the trial has been registered on the Chinese Clinical Trial Registry (Registration Number: ChiCTR-IPR-17011875). According to the World Health Helsinki Declaration, each patient signed an informed consent form before the trial began. The flow diagram of the study is shown in Figure 1.

### Participants

Participants were recruited using posters, by distributing leaflets, advertising in newspapers and other media, as well as by obtaining hospitalization information from Lianyungang hospital affiliated with the Xuzhou Medical University. The inclusion criteria were as follows: (1) definite diagnosis of primary PD in line with the PD diagnostic guidelines of the 2013 European Neurology Alliance (Berardelli et al., 2013); (2) Hoehn-Yahr (HY) grade 1–3 and independent ability to walk; (3) drug treatment not adjusted during the last week before the trial start; (4) normal hearing, visual, and cognitive ability. The medication was not changed during the Baduanjin exercise program, and no other physical exercise was accepted. The exclusion criteria were as follows: (1) various secondary Parkinson’s syndromes (e.g., trauma, drug-induced, neoplastic, vascular, toxic, hydrocephalus, etc.) and Parkinson’s superposition syndrome; (2) previous bone and joint disease or cerebrovascular disease, and defective walking or balance; (3) heart dysfunction, severe hypertension, and other serious medical diseases; (4) non-cooperating patients during the examination and training. For participants who withdrew from the trial, a questionnaire survey was conducted, and the reasons were recorded in detail.

### Intervention

Based on conventional medical treatment, each patient received Baduanjin exercise training according to the “Health Qigong Baduanjin Standard” enacted by the General Administration of Sports in 2003. Eight sections of movements were performed by the participants during the exercise phase. Each session was guided by a sports scientist with experience in Baduanjin exercises, aided by two professional Chinese medicine rehabilitation specialist. Patients completed the sessions at the Department of Neurology or Rehabilitation every morning from 8:00 to 9:00. Subjects completed the supervised Baduanjin exercise program for about 36 min per session, once a day, for 3 weeks. Practice involved joint stretching for 5 min, 3 min of rest, 12 min of exercise practiced 2 times, and then 4 min of tapping and relaxing exercise. The researcher ensured the accuracy of the participant’s performance during the Baduanjin exercise, and two supervisors corrected the movement and gestures. Researchers recorded the subjects’ heart rate and blood pressure before and after training. At the end, they gave the activity log and self-report results to the project administrator. The adverse events occurring during the study and their causes were also recorded, and the incidence of adverse events was calculated as an evaluation index.

### Intelligent Device for Energy Expenditure and Activity Gait and Balance-Check^®^ Tests

The secondary outcomes were IDEEA gait and Balance-check^®^ balancer test scores. IDEEA (MiniSun, Fresno, CA) is a new device for assessing physical activity (Werner et al., 2012) and can be used to analyze gait, distance, power, speed, work, and energy consumption. IDEEA was shown to accurately measure postures and limb movements, as well as walking and running speeds (Kwon et al., 2010). Each patient was asked to walk straight for 10 m, back and forth, at a normal natural speed. A total of 15 gait parameters were measured in the study, including Single-limb Support (SLS; ms), Double-limb Support (DLS; ms), SLS/DLS (%), etc. The Balance-check^®^ Balance Detection and Rehabilitation System (by Dr. Wolff, Germany) is a balanced detection and training system with biofeedback (Yuan and Jia-Hong, 2018). We used the Balance Test mode, in which each test lasts for 1 min, with a difficulty level of 5 (medium difficulty). In this study, we measured four test indexes, including score, maximum speed, minimum speed, and grade. The higher the score, the better the dynamic balance ability. Grade is an intuitive index to evaluate dynamic balance and is divided into the following six categories: very good (1–1.5), good (1.6–2.5), satisfactory (2.6–3.5), sufficient (3.6–4.5), insufficient (4.6–5), and unsatisfactory (5.6–6).

### Testing Procedure

Each participant was tested in the medication ON-state (within 1 h after taking the anti-PD drug) and performed the exercise at the same time of day.

### Statistical Analyses

GraphPad Prism 5 software was used for mapping analysis, and SPSS 22.0 software was used for statistical analysis. Measured data are presented as mean ± standard deviation. A paired sample *t*-test was undertaken to compare the scores at baseline and after the intervention, and *P* ≤ 0.05 was considered statistically significant.

### Patient and Public Involvement

During the application and implementation of the trial, we formed a Patient and Public Participation (PPI) group, and we continue to work closely with them. PPI has participated and communicated through face-to-face interviews, via e-mail, WeChat and telephone, and patients were recruited through WeChat and newspapers. Only those with mild to moderate Parkinson’s disease who can fully cooperate with the completion of the training program and related examinations were included in the training. Two PPI representatives performed the recommended exercise program at home, so they provided feedback on the usefulness and effectiveness of the program. The PPI team reviewed the PILs, consent forms, and posters advertising the trial. A regular interview schedule was developed with the help of PPI representatives.

## Results

### General Information for All Participants

The study was commenced in May 2017 and ended in September 2018. A total of 31 participants were enrolled, and 4 dropped out of the study. Finally, 27 participants completed the trial, including 15 men (55.6%). The average age, height, and body weight were 65.37 ± 7.47 years, 164.00 ± 7.82 cm, and 66.46 ± 11.74 kg, respectively. The duration of the disease was 6.63 ± 2.27 years, and medication was taken for an average of 5.37 ± 2.19 years. The HY stage was 1.85 ± 0.59. Eight (29.6%) patients had hypertension and five (18.5%) had diabetes (Table 1). No serious injuries, such as falls, or any accidental injuries occurred during exercise training.

**TABLE 1:** Basic information of Parkinson’s disease patients.

Characteristics	*n* = 27 Mean (*SD*) or Number (proportion)
Age (years)	65.37(7.45)
Sex (men)	15(55.6%)
Height (cm)	164(7.8)
Weight (kg)	66.46(11.74)
Years with PD (years)	6.63(2.27)
History of medication (years)	5.37(2.19)
Hoehn and Yahr, stage	1.85(0.59)
Patients with hypertension (%)	8(29.6%)
Patients with diabetes (%)	5(18.5%)

### Modified Unified Parkinson’s Disease Rating Scale and Non-motor Symptoms Scale Scores

Changes in MDS-UPDRS and NMSS scores from baseline to 3 weeks post-training served as the primary outcome. The total score on the MDS-UPDRS was significantly lower after completing Baduanjin exercise in PD patients (*t* = 4.669, *P* ≤ 0.001). Scores in three of the four MDS-UPDRS parts were significantly lower after the exercise program (part I: *t* = 5.805, *P* ≤ 0.001; part II: *t* = 5.234, *P* ≤ 0.001; part III: *t* = 3.274, *P* = 0.003), but no significant difference was observed for the score of part IV (*t* = 1.000, *P* = 0.327). In MDS-UPDRS part I, non-motor symptoms significantly improved, including depressed mood (*t* = 3.606, *P* = 0.001), anxious mood (*t* = 3.574, *P* = 0.001), apathy (*t* = 3.911, *P* = 0.001), nighttime sleep problems (*t* = 3.698, *P* = 0.001), daytime sleepiness (*t* = 2.563, *P* = 0.017), lightheadedness on standing (*t* = 2.126, *P* = 0.043), and fatigue (*t* = 3.606, *P* = 0.001). The same was true for items of MDS-UPDRS part II such as eating (*t* = 2.563, *P* = 0.017), dressing (*t* = 4.000, *P* ≤ 0.001), hygiene (*t* = 4.561, *P* ≤ 0.001), turning in bed (*t* = 4.914, *P* ≤ 0.001), getting out of bed, car, or deep chair (*t* = 2.563, *P* = 0.017), and walking and balance (*t* = 2.726, *P* = 0.011; Table 2).

**TABLE 2:** Comparison of Modified Unified Parkinson’s Disease Rating Scale (MDS-UPDRS) and Non-Motor Symptoms Scale (NMSS) scale score before and after Baduanjin exercise in Parkinson’s patients.

Scale	*n* = 27		*P*
	Before	After	
MDS-UPDRS total score	57.78 ± 13.95	51.30 ± 13.99	≤ 0.001
MDS-UPDRS part-I	12.44 ± 4.25	9.59 ± 5.03	≤ 0.001
Cognitive impairment	0.37 ± 0.57	0.33 ± 0.48	0.327
Hallucinations and psychosis	0.22 ± 0.42	0.15 ± 0.36	0.327
Depressed mood	1.48 ± 1.05	1.04 ± 0.94	0.001
Anxious mood	1.44 ± 1.05	0.96 ± 0.98	0.001
Apathy	1.52 ± 1.09	1.15 ± 1.03	0.001
Features of dopamine	0.15 ± 0.36	0.15 ± 0.36	1.000
Nighttime sleep problems	1.52 ± 0.85	1.11 ± 0.89	0.001
Daytime sleepiness	1.11 ± 0.64	0.85 ± 0.72	0.017
Pain and other sensations	1.00 ± 0.78	0.93 ± 0.87	0.425
Urinary problems	0.59 ± 0.69	0.44 ± 0.70	0.161
Constipation problems	0.89 ± 0.64	0.81 ± 0.68	0.161
Lightheadedness on standing	0.56 ± 0.64	0.41 ± 0.50	0.043
Fatigue	1.59 ± 0.70	1.26 ± 0.81	0.001
MDS-UPDRS part-II	15.44 ± 5.15	13.04 ± 4.56	≤ 0.001
Speech	0.96 ± 0.76	0.96 ± 0.81	1.000
Salivation and drooling	1.26 ± 0.86	1.07 ± 0.83	0.096
Chewing and swallowing	0.48 ± 0.70	0.52 ± 0.70	0.713
Eating tasks	1.11 ± 0.58	0.85 ± 0.53	0.017
Dressing	1.44 ± 0.58	1.00 ± 0.68	≤ 0.001
Hygiene	1.59 ± 0.64	1.15 ± 0.66	≤ 0.001
Handwriting	0.85 ± 0.46	0.89 ± 0.58	0.663
Doing hobbies and other activities	1.15 ± 0.36	1.07 ± 0.55	0.490
Turning in bed	1.81 ± 0.62	1.33 ± 0.73	≤ 0.001
Tremor	1.63 ± 0.79	1.56 ± 0.89	0.425
Getting out of bed car or a deep chair	1.41 ± 0.64	1.15 ± 0.66	0.017
Walking and balance	1.48 ± 0.58	1.26 ± 0.66	0.011
Freezing	0.26 ± 0.45	0.22 ± 0.42	0.663
MDS-UPDRS part-III	26.85 ± 7.05	24.85 ± 6.91	0.003
MDS-UPDRS part-IV	3.04 ± 2.48	2.97 ± 2.34	0.327
NMSS total score	57.82 ± 16.65	49.56 ± 15.66	≤ 0.001

Scores on the NMSS were also significantly lower after than before exercise training (*t* = 4.815, *P* ≤ 0.001; Table 2).

### Intelligent Device for Energy Expenditure and Activity Gait and Balance-Check^®^ Test

A total of 15 gait parameters in IDEEA were included in the study, including SLS (ms), DLS (ms), SLS/DLS (%), swing duration (ms), step duration (ms), cycle duration (s), pulling acceleration (G), swing power (G), ground impact (G), foot fall, push off, speed (m/min), cadence (steps/min), step length (m), and stride length (m). Among these parameters, step duration (*t* = 2.289, *P* = 0.030) and cycle duration (*t* = 2.181, *P* = 0.038) were significantly lower after Baduanjin exercise. Other gait parameters demonstrated no significant differences pre-and post-training (Table 3).

**TABLE 3:** Comparison of Intelligent Device for Energy Expenditure and Activity (IDEEA) gait and Balance-check^®^: Data before and after Baduanjin exercise in Parkinson’s patients.

Data	*n* = 27		*P*
	Before	After	
**IDEEA gait data**			
Single support (ms)	452.55 ± 100.80	461.94 ± 103.82	0.678
Double support (ms)	178.33 ± 100.02	159.49 ± 88.13	0.462
SLS/DLS (%)	223.77 ± 80.81	186.63 ± 59.67	0.051
Swing duration (ms)	647.22 ± 401.73	574.19 ± 261.73	0.284
Step duration (ms)	747.35 ± 239.39	645.90 ± 156.65	0.030
Cycle duration (s)	1.48 ± 0.43	1.30 ± 0.32	0.038
Pulling Accel. (G)	0.79 ± 0.36	0.88 ± 0.25	0.188
Swing power (G)	0.47 ± 0.18	0.46 ± 0.12	0.855
Ground impact (G)	0.83 ± 0.31	0.89 ± 0.21	0.163
Foot fall	2.14 ± 0.87	2.30 ± 0.58	0.243
Push off	20.67 ± 7.60	20.88 ± 9.87	0.920
Speed (m/min)	40.29 ± 11.97	41.31 ± 9.53	0.582
Cadence (steps/min)	93.74 ± 19.69	97.70 ± 15.32	0.318
Step length (m)	0.45 ± 0.05	0.46 ± 0.077	0.599
Stride length (m)	0.77 ± 0.14	0.84 ± 0.14	0.141
**Balance-check^®^ data**			
Balance test score	25375.15 ± 9628.60	29252.70 ± 9086.54	0.041
Minimum speed	1.68 ± 2.39	1.29 ± 1.85	0.221
Maximum speed	7.27 ± 6.69	6.16 ± 7.18	0.477
Grade	2.39 ± 1.35	1.69 ± 1.07	0.002

*SLS, Single-limb Support; DLS, Double-limb Support.*

In the Balance-check^®^ test, we used the balance test mode. Test data included the following four items: total score, maximum speed, minimum speed, and grade. Among these indicators, we found statistically significant differences in the total score (*t* = -2.147, *P* = 0.041) and grade (*t* = 3.432, *P* = 0.002). After exercise, the total score of patients was higher, and the grade was better. No significant differences were observed for maximum and minimum speed after exercise training (Table 3).

## Discussion

With the aging of the population, the prevalence of PD is on the rise (Dorsey et al., 2007). PD leads to deterioration of balance and gait, leading to falls and injuries and a decline in physical activity, and thus, leading to a decline in the quality of life (Dontje et al., 2013). At present, there is no radical treatment for PD. The main means of clinical intervention is drug therapy, aiming to minimize the clinical symptoms of patients and maintain their ability for independence and quality of life as long as possible (Chaudhuri et al., 2017). Rehabilitation therapy has been considered to improve the motor and non-motor symptoms of PD patients, improve the quality of daily life, postpone medication or reduce the dose of medication, and promote postoperative functional recovery (Abbruzzese et al., 2016). In addition, there are some unconventional rehabilitation strategies, including music therapy, dance therapy, martial arts therapy (Tai Chi), motor imagery, action observation therapy, exergaming, and robot-assisted training (Li et al., 2005; Abbruzzese et al., 2016). It is important to find an easy-to-learn, low-cost, and effective intervention to improve the symptoms of these patients.

Many studies have focused on the improvement of motor symptoms in patients with PD. For example, Dipasquale et al. (2017) randomly allocated 40 patients with PD of HY stage 2 to the following two groups: one followed a general exercise (GE) program, and one followed a physical therapy (PT) program. Outcome indicators included the Functional Independence Measure, Hamilton Rating Scale, Timed Up & Go test, and Unified Parkinson’s Disease Rating Scale (UPDRS) score, which were all significantly better for the PT group when compared with the GE group at the end of treatment. Thus, physiotherapy seems to be more effective than GE in patients with HY stage II PD. In our trial, the total and sub-scores on the MDS-UPDRS and its parts II and III were lower after exercise, but there was no significant difference in part IV. As scores in MDS-UPDRS part II items like eating, dressing, hygiene, turning in bed, walking and balance, and getting out of bed, car, or deep chair, significantly improved after training, and since we included patients of HY stage 1–3, we conclude that Baduanjin exercise might have beneficial effects on the motor symptoms of such patients with mild to moderate PD.

A previous review explored studies addressing the effects of exercise and physical activity on autonomic dysfunction, cognitive impairment, and sleep disorders in PD (Amara and Memon, 2018). Nevertheless, effective motor interventions for these symptoms, as well as the mechanisms of exercise-induced changes, and the best ways to monitor treatment responses remain unclear. Some existing studies have shown that exercise is an effective way to improve non-motor symptoms in patients with PD. Consistently, we found that scores both on MDS-UPDRS part-I and NMSS significantly improved after exercise. Specifically, non-motor symptoms of MDS-UPDRS part-I, including depressed mood, anxious mood, apathy, nighttime sleep problems, daytime sleepiness, lightheadedness on standing, and fatigue, significantly improved with Baduanjin training. Thus, we conclude that Baduanjin exercise has beneficial effects on non-motor symptoms in patients with mild to moderate PD.

Many studies have shown that rehabilitation, aerobic exercise, and many traditional Chinese exercises can significantly improve balance in patients with PD. For example, Tsang randomized 185 patients with PD (HY stage 1–4) in groups following Tai Chi, resistance training (8–10 leg muscle strengthening exercises), or just stretching involving the upper and lower limbs (Tsang, 2013). Tsang showed that the maximum offset was significantly higher in the Tai Chi group than in the resistance and stretching groups, and that direction control, stride length, and functional range significantly improved. Moreover, the peak torque, rise and fall test time, and UPDRS part-III score regarding knee flexion and extension were significantly improved after Tai Chi training. Therefore, Tsang concluded that Tai Chi training effectively alleviates balance deficits in patients with mild to moderate PD. In our trial, we found statistically significant improvements in the total score and grade of the Balance-check^®^ test after exercise. Thus, we conclude that Baduanjin exercise is also beneficial for improving balance in these patients.

Other types of physical exercise have also been shown to improve balance in patients with PD. For example, Cheng et al. examined the effects of a 4–6-week curved-walking training program in patients with PD (Cheng et al., 2017). The authors randomized 24 patients in control and training groups and showed that both the performance in curved-walking and gait freezing, as measured by the freezing of gait questionnaire, were significantly improved after training. Secondary outcomes, including straight-walking performance (speed, cadence, and step length), functional gait assessment, Timed Up and Go test score, UPDRS part-III score, and quality of life were also improved in the group that received training. In our trial, we measured a total of 15 gait parameters, among which step and cycle duration were significantly improved after the Baduanjin exercise program. We conclude that Baduanjin exercise positively affects gait in patients with mild to moderate PD. Xiao et al. (2021) analyzed 8 randomized clinical trial studies on Baduanjin, and they believed that Baduanjin can improve the balance function in middle-aged and elderly people and reduce the fall rate.

In ancient China, Baduanjin was originally used to help soldiers recover from bodily injuries (Zou et al., 2017a). Similar to other traditional Chinese health-promoting activities, like Tai Chi and Wuqinxi, Baduanjin is based on the body’s own physical activities, breathing, and psychological adjustment and is important for enhancing physical fitness (Chen et al., 2017). A growing number of studies have shown that regular Baduanjin training can improve physical and mental outcomes in the elderly, such as improving blood lipid metabolism, lowering blood pressure, reducing depression and anxiety, and improving sleep quality and physical flexibility (Mei et al., 2012; Chen et al., 2016, 2018; Tao et al., 2017). Thus, Baduanjin has become a popular community activity for the elderly in China. Baduanjin involves eight blocks of simple, slow, and relaxing movements, which need to be practiced on both sides of the body while incorporating deep-temporal breathing, meditation, and musculoskeletal stretching and relaxation (Zou et al., 2017b). Contrary to other more complex forms of training, like Tai Chi, Baduanjin is less physically and cognitively demanding. Therefore, this exercise is easier to learn and more suitable for older adults, especially those with PD. Carvalho et al. (2021) used the TeraPlus platform to supervise two patients with early to mid-stage PD (with a disease course of at least 3 years) to perform Baduanjin Qigong exercises at home twice a week (over 8 weeks). The results showed that both participants had significant improvements in gait speed, balance, and perceived health-related quality of life. However, this study design had obvious limitations because the results were only obtained from two participants. Data from more participants are required for more convincing results.

This study has certain limitations. First, the results were only based on the data of 27 participants. In the future, a larger sample size will be used. In addition, we did not set up a randomized control group, and hence cannot completely exclude the influence of other interfering factors on the experimental results. Finally, our study did not analyze whether the cognitive function will affect the degree of cooperation of patients, which may also affect the results of the experiment.

Baduanjin has a range of practical advantages as it is a low-cost exercise that does not require equipment. It can be carried out at anytime and anywhere and is extremely easy to learn. Baduanjin can also be integrated into existing treatments as part of the patients’ rehabilitation. Because of its simplicity, Baduanjin can be used as part of the patient’s family exercise routine, which can be conducive for the rehabilitation of PD patients with important social and economic benefits.

## Conclusion

Baduanjin exercise is an acceptable, feasible, and safe intervention with beneficial effects on the non-motor symptoms, balance, and gait, thus promoting the performance of daily activities in patients with PD. Application of this simple, traditional exercise is recommended for patients with PD as a complementary treatment for improving their quality of life. Future randomized controlled trials, with longer intervention periods, and larger sample sizes are needed to establish the effects of Baduanjin on the rehabilitative outcomes in PD patients.

## Data Availability Statement

The original contributions presented in the study are included in the article/supplementary material, further inquiries can be directed to the corresponding author/s.

## Ethics Statement

The studies involving human participants were reviewed and approved by the Ethics Committee of Nanjing Medical University. The patients/participants provided their written informed consent to participate in this study.

## Author Contributions

ZC and SSD played a critical role in conceptualizing this study. YW, HW, and SYD collected and analyzed all the data. XL, JZ, and YW prepared the manuscript. All authors provided critical feedback on the manuscript. All authors have read and approved the submitted manuscript. The manuscript has not been submitted or published elsewhere in whole or in part.

## Conflict of Interest

The authors declare that the research was conducted in the absence of any commercial or financial relationships that could be construed as a potential conflict of interest.

## Publisher’s Note

All claims expressed in this article are solely those of the authors and do not necessarily represent those of their affiliated organizations, or those of the publisher, the editors and the reviewers. Any product that may be evaluated in this article, or claim that may be made by its manufacturer, is not guaranteed or endorsed by the publisher.
